# Assessment of association between lower ureteric excision technique and oncological outcomes for upper urinary tract urothelial carcinoma: retrospective analysis from the Scottish Renal Cancer Consortium

**DOI:** 10.1007/s00345-023-04283-5

**Published:** 2023-01-24

**Authors:** James Peter Blackmur, Etienne Chew, Matthew Trail, Katie Brodie, Nicola Santoni, Flora Rodger, David Hamilton, Fortis Gaba, Sophie Randall, Sarika Nalagatla, Brian Little, Khalid Janjua, Clare Sweeney, Andrew Martindale, Khaver Qureshi, Antony Riddick, Kevin O’Connor, S. Alan McNeill, Simon Phipps, Mark L. Cutress, Edward A. A. Mains, Ian Dunn, Sarah Reid, Grant D. Stewart, Gavin Lamb, Muhammad Zeeshan Aslam, Steve Leung, Ross Clark, Ian Wilson, Grenville Oades, Alexander Chapman, Alexander Laird

**Affiliations:** 1 Institute of Genetics and Cancer, University of Edinburgh, Edinburgh, UK; 2 Department of Urology, NHS Lothian, Edinburgh, UK; 3 College of Medicine, University of Edinburgh, Edinburgh, UK; 4 Department of Urology, NHS Fife, Kirkcaldy, UK; 5 Department of Urology, NHS Highland, Inverness, UK; 6 Department of Urology, NHS Ayrshire and Arran, Ayr, UK; 7 Department of Urology, NHS Tayside, Dundee, UK; 8 Department of Urology, NHS Greater Glasgow and Clyde, Glasgow, UK; 9 College of Medicine, University of Glasgow, Glasgow, UK; 10 Department of Urology, NHS Lanarkshire, Airdrie, UK; 11 Department of Urology, Addenbrooke’s Hospital, Cambridge, UK; 12 Department of Urology, Cork University Hospital, Cork, Republic of Ireland; 13 Department of Surgery, University of Cambridge, Cambridge, UK; 14 Department of Urology, NHS Forth Valley, Larbert, UK

**Keywords:** Upper tract urothelial carcinoma, UTUC, Nephroureterectomy, Surgical technique, Recurrence-free survival

## Abstract

**Purpose:**

Nephroureterectomy(NU) remains the gold-standard surgical option for the management of upper urinary tract urothelial carcinoma(UTUC). Controversy exists regarding the optimal excision technique of the lower ureter. We sought to compare post-UTUC bladder tumour recurrence across the Scottish Renal Cancer Consortium(SRCC).

**Methods:**

Patients who underwent NU for UTUC across the SRCC 2012–2019 were identified. The impact of lower-end surgical technique along with T-stage, N-stage, tumour location and focality, positive surgical margin, pre-NU ureteroscopy, upper-end technique and adjuvant mitomycin C administration were assessed by Kaplan–Meier and Cox-regression. The primary outcome was intra-vesical recurrence-free survival (B-RFS).

**Results:**

In 402 patients, the median follow-up was 29 months. The lower ureter was managed by open transvesical excision in 90 individuals, transurethral and laparoscopic dissection in 76, laparoscopic or open extra-vesical excision in 31 and 42 respectively, and transurethral dissection and pluck in 163. 114(28.4%) patients had a bladder recurrence during follow-up. There was no difference in B-RFS between lower-end techniques by Kaplan–Meier (*p* = 0.94). When all factors were taken into account by adjusted Cox-regression, preceding ureteroscopy (HR 2.65, *p* = 0.001), lower ureteric tumour location (HR 2.16, *p* = 0.02), previous bladder cancer (HR 1.75, *p* = 0.01) and male gender (HR 1.61, *p* = 0.03) were associated with B-RFS.

**Conclusion:**

These data suggest in appropriately selected patients, lower ureteric management technique does not affect B-RFS. Along with lower ureteric tumour location, male gender and previous bladder cancer, preceding ureteroscopy was associated with a higher recurrence rate following NU, and the indication for this should be carefully considered.

**Supplementary Information:**

The online version contains supplementary material available at 10.1007/s00345-023-04283-5.

## Introduction

Upper urinary tract urothelial cell carcinoma (UTUC) is rare, accounting for only 5–10% of cases of UC [1, 2] however is a biologically aggressive disease with a high potential for recurrence and death [3]: 5-year cancer-specific survival (CSS) rates have been described as 86% with ≤ T1 stage disease, whereas 32% for ≥ T3 stage disease [4]. Radical nephroureterectomy (NU) with en-bloc excision of the ureteric orifice from the bladder wall is the standard curative surgical treatment [1]. Although open nephroureterectomy (ONU) remains the gold standard for high-risk UTUC [1], it is associated with significant morbidity [5]. Laparoscopic nephroureterectomy (LNU) and robotic nephroureterectomy (RNU) have increasingly been used as minimally invasive alternatives, with studies suggesting equivalent oncological outcomes (2- and 5-year recurrence-free survival (RFS), CSS and overall survival (OS) [3, 6–12]).

Controversy exists regarding the optimal excision technique for the management of the lower ureter. While several techniques have been considered to simplify lower ureter resection, including the pluck technique, stripping and transurethral resection of the intramural ureter, these techniques may not have equivalent oncological outcomes to complete bladder cuff excision [13–15]. Of the studies comparing outcome by different lower-end surgical techniques, there has been little heterogeneity in the techniques used [14, 16]. We sought to compare the impact of lower ureteric excision technique along with other patient, tumour and surgical factors on oncological outcomes across the Scottish Renal Cancer Consortium (SRCC).

## Methods

Patients were identified who underwent nephroureterectomy for UTUC across the SRCC between 2012 and 2019. Nine urology departments offer UTUC management to the Scottish population of approximately 5.5 million people; 8 centres took part in this study.

**Fig. 1:**
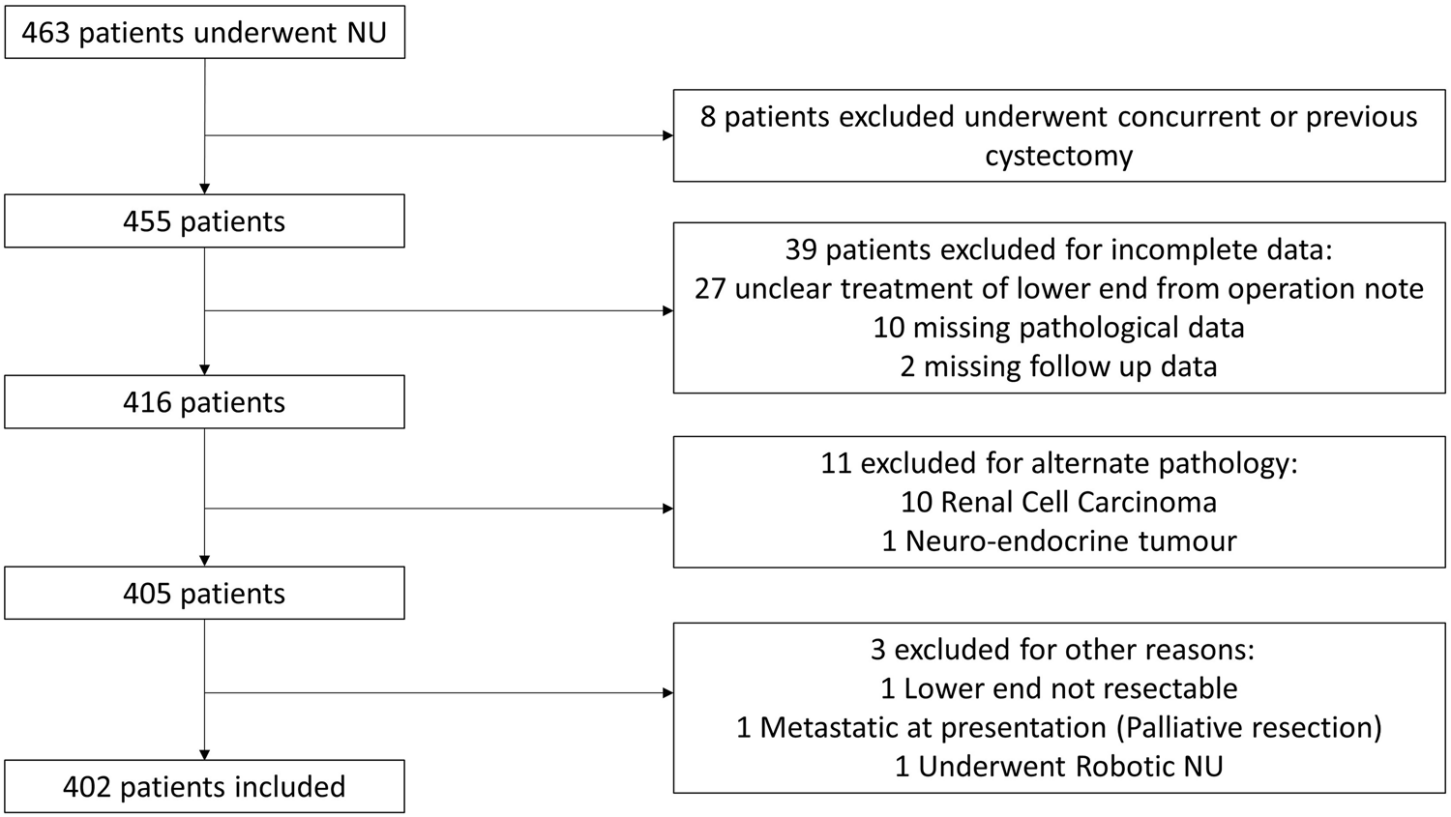
Study Consort diagram

Patients with previous or concurrent cystectomy were excluded as this removed possible bladder recurrence. In addition, patients with incomplete data were excluded from the analysis (Fig. 1).

All patients were managed by a local multi-disciplinary team (MDT) according to local protocols. Surgical technique selected was at the discretion of the institution and surgeon. “Upper-end” (i.e., kidney and upper ureter) dissection was undertaken by laparoscopic or open approach. No centre in Scotland was undertaking robot-assisted NU routinely during this time period (one patient underwent RNU at the end of the study, and was excluded from analyses for this reason). Five definitions for lower-end management were agreed prior to data collection by surgical consultants in the SRCC, as detailed in Table 1. Nodal dissection was undertaken where there was suspicion of nodal involvement on pre-operative computed tomography (CT) scan, as per contemporaneous guidelines. Tumour grade, stage (standardised to 2018 8^th^ edition of TNM) and presence of positive surgical margin were recorded from reports produced by specialist Consultant Uropathologist prior to local MDT review. Patients were followed up after a review of final pathology results at the MDT meeting, with cystoscopic and radiological surveillance as per contemporaneous EAU Guidelines.

**Table 1:** Description of lower ureter management techniques. In cases when the tumour involved the mid-ureter or above, the principles of early ureteric mobilisation and ligation below the level of the tumour during the intra-abdominal phase of the procedure were employed whenever possible

Technique	Description
Open transvesical excision	Open dissection of the distal ureter to bladder. Bladder then opened via a separate incision and intra-mural ureter controlled by retrograde dissection of bladder cuff around ureteric orifice after suture ligation
Combined transurethral and laparoscopic dissection	Transurethral retrograde resection of ipsilateral ureteric orifice and intramural ureter using diathermy loop until fat encountered. Open ureteric orifice sealed with diathermy and resected chips of ureter retrieved with Ellick’s evacuator (and sent for Pathological assessment) and bladder catheterised. Laparoscopic dissection of kidney and antegrade ureteric dissection to resected end
Laparoscopic extra-vesical excision	Laparoscopic dissection of the distal ureter, with distal (intramural) ureter controlled by antegrade dissection of bladder cuff around ureteric orifice
Open extra-vesical excision	Open dissection of the distal ureter, with distal (intramural) ureter controlled by antegrade dissection of bladder cuff around ureteric orifice
Transurethral dissection and pluck	Transurethral dissection around ipsilateral ureteric orifice using Collin’s knife. Retrograde dissection around intra-mural ureter until fat encountered. Ureteric orifice is sealed with diathermy before the bladder is catheterised. Kidney and proximal ureteric dissection, with distal ureter excised in continuity by a combination of blunt and sharp antegrade dissection until the free end encountered

The primary outcome was intra-vesical (bladder) recurrence-free survival (B-RFS). Secondary outcomes were extra-vesical pelvic recurrence-free survival (P-RFS), distant metastatic recurrence-free survival (M-RFS) and overall survival (OS).

The impact of surgical technique for the lower ureter on B-RFS was assessed by Kaplan–Meier method and log-rank test. The impact of surgical technique for the lower ureter on primary and secondary outcomes was also assessed by unadjusted and adjusted multivariable Cox regression. Covariables included in the model were T-stage, N-stage, pre-operative diagnostic ureteroscopy, whether tumour was uni- or multi-focal, location of the most distal tumour, surgical technique for the upper-end, whether the surgical margin was positive, administration of adjuvant intra-vesical chemotherapy (mitomycin C), gender, age, past history of bladder cancer and operating centre were included in the model. Adjuvant intra-vesical mitomycin C was provided prior to the removal of the catheter at 10–14 days post-operatively, in line with the protocol used within the ODMIT-C study [17]. Variables were assessed for collinearity and excluded if this was significant, with a high correlation (Pearson rho > 0.5). Factors included in the models were on the basis of previous studies identifying association with tumour recurrence [1, 4, 13, 14, 16]. In addition, the associations of T-stage, tumour location, upper-end and lower-end surgical technique with positive surgical margin were assessed using logistic regression.

Continuous variables are reported by median and inter-quartile range (IQR). Discrete variables are reported by number and percentage. *P* values < 0.05 were considered significant. All statistical analysis was performed using R [18] and RStudio [19]. Kaplan–Meier, Cox-regression and logistic regression were performed with the *survival* [20], *survminer* [21] and *finalfit* [22] packages. Data were collected by individuals within each participating centre according to local Caldicott Guardian clearance. Patient data were anonymised before collation centrally prior to analysis.

## Results

### Patient characteristics

402 patients were included from 8 centres (Fig. 1). Demographic, clinical and pathological data are presented in Table 2. Median follow-up was 29 months (IQR 16–54 months). 184 (45.8%) patients had a recurrence during follow-up: 114 (28.4%) with intra-vesical recurrence, 29 (7.2%) with extra-vesical pelvic recurrence and 100 (24.9%) patients developed distant metastatic disease. 142 (35.3%) patients died during follow-up, of which 91 (22.6%) patients died secondary to UC.

**Table 2:** Demographic, Tumour, Pathological and Surgical data of individuals included in the study

Factor	Description	
Patient		
Age	Median (IQR), years	71 (64–77)
Gender	Female	153 (38.1%)
	Male	249 (61.9%)
Smoking status	Non	126 (31.3%)
	Ex	33 (8.2%)
	Current	172 (42.8%)
	Unknown	71 (17.7%)
Previous bladder cancer	No	309 (76.9%)
	Yes	93 (23.1%)
Tumour		
Focality	Unifocal	362 (90.0%)
	Multifocal	40 (10.0%)
Most distal tumour location	Calyces/renal pelvis	145 (36.1%)
	Upper ureter	88 (21.9%)
	Mid ureter	39 (9.7%)
	Lower ureter	130 (32.3%)
Pathological		
T-stage	pTa	178 (44.3%)
	pT1	57 (14.2%)
	pT2	42 (10.4%)
	pT3	105 (26.1%)
	pT4	16 (4.0%)
	CIS	4 (1.0%)
Grade	1	14 (3.5%)
	2	195 (48.5%)
	3	189 (47.0%)
	CIS	4 (1.0%)
N-stage	pN0/Nx	378 (94.0%)
	pN +	24 (6.0%)
Positive surgical margin	No	359 (89.3%)
	Yes	43 (10.7%)
Surgical		
Pre-operative URS	No	101 (25.1%)
	Yes	301 (74.9%)
Upper-end surgical technique	Laparoscopic	350 (87.1%)
	Laparoscopic converted to open	7 (1.7%)
	Open	45 (11.2%)
Lower-end surgical technique	Open transvesical excision	90 (22.4%)
	Combined transurethral & laparoscopic dissection	76 (18.9%)
	Laparoscopic extra-vesical excision	31 (7.7%)
	Open extra-vesical excision	42 (10.4%)
	Transurethral dissection and pluck	163 (40.5%)
Post-operative intra-vesical Mitomycin C	No	323 (80.3%)
	Yes	79 (19.7%)

Data represent *n* (%) unless otherwise stated

### Association of patient, tumour and surgical factors with bladder-recurrence free survival

There was no difference in B-RFS between lower-end techniques by KM (*p* = 0.94, Supp Fig. 1). There was a significant correlation between T-stage and tumour grade (Pearson rho 0.62, *p* < 2.2e-16). Grade was therefore not included in Cox regression models to avoid collinearity. The presence of a multifocal ureteric tumour was weakly correlated with a history of previous bladder cancer (Pearson rho 0.13, *p* = 0.007); these factors were both included in models. T-stage was weakly correlated with N-stage (Pearson rho 0.23, *p* = 3.55e-6); these factors were both included in models.

On unadjusted Cox-regression analysis, pTis stage, lower ureteric tumour location, preceding ureteroscopy and previous bladder cancer were associated with B-RFS (Table 3). When all factors above were taken into account by adjusted Cox-regression, lower ureteric tumour location, preceding ureteroscopy, previous bladder cancer and male gender were associated with increased risk of B-RFS (Table 3). The substitution of grade for stage in the adjusted Cox-regression analysis did not alter the factors predictive of outcome (data not shown).

**Table 3:** Association of Patient, tumour or surgical factors with B-RFS in multivariable Cox-regression analysis

Variable	*N*	Unadjusted analysis			Adjusted analysis		
		HR	95% CI	*P* value	HR	95% CI	*P* value
T stage							
pTa	178	Ref	–	–	Ref	–	–
pT1	57	1.40	0.85–2.30	0.18	1.46	0.87–2.44	0.15
pT2	42	1.22	0.68–2.20	0.51	0.93	0.49–1.74	0.81
pT3	105	0.72	0.42–1.21	0.21	0.66	0.37–1.15	0.14
pT4	16	0.27	0.04–1.92	0.19	0.23	0.03–1.75	0.16
pTis	**4**	**3.63**	**1.13–11.60**	**0.03**	1.96	0.56–6.87	0.29
N stage							
pN0/Nx	378	Ref	–	–	Ref	–	–
pN +	24	0.65	0.21–2.06	0.47	0.85	0.05–3.50	0.41
Focality							
Unifocal	360	Ref	–	–			
Multifocal	40	1.60	0.93–2.75	0.09	1.30	0.68–2.48	0.42
Most distal tumour location							
Renal pelvis/calyces	145	Ref	–	–	Ref	–	–
Upper ureter	88	1.23	0.72–2.10	0.46	1.34	0.73–2.45	0.35
Mid ureter	39	1.58	0.84–2.96	0.15	1.45	0.71–2.96	0.30
Lower ureter	130	**1.90**	**1.21–2.99**	**0.006**	**2.16**	**1.16–3.99**	**0.02**
Ureteroscopy preceding NU							
No URS	101	Ref	–	–	Ref	–	–
URS	301	**2.54**	**1.47–4.37**	** < 0.001**	**2.65**	**1.50–4.69**	**0.001**
Upper-end surgical technique							
Laparoscopic	350	Ref	–	–	Ref	–	–
Laparoscopic converted to open	7	1.21	0.38–3.83	0.74	1.50	0.41–5.47	0.54
Open	45	0.86	0.44–1.71	0.68	0.93	0.42–2.06	0.85
Lower-end surgical technique							
Open transvesical excision	90	Ref	–	–	Ref	–	–
Combined transurethral & lap dissection	76	0.81	0.44–1.49	0.50	1.57	0.67–3.68	0.31
Laparoscopic extra-vesical excision	31	0.87	0.28–2.00	0.74	1.21	0.44–3.34	0.71
Open extra-vesical excision	42	1.08	0.56–2.10	0.81	1.49	0.63–3.51	0.37
Transurethral dissection and pluck	163	0.94	0.59–1.50	0.79	1.43	0.74–2.75	0.28
Positive surgical margin							
No	359	Ref	–	–	Ref	–	–
Yes	43	1.13	0.59–2.18	0.70	1.38	0.66–2.86	0.39
Mitomycin C installation							
No	323	Ref	–	–	Ref	–	–
Yes	79	1.36	0.89–2.08	0.15	1.56	0.83–2.93	0.17
Gender							
F	153	Ref	–	–	Ref	–	–
M	249	1.32	0.89–1.94	0.16	**1.61**	**1.06–2.45**	**0.03**
Age at operation							
Mean (SD)	69.9 (9.9)	1.00	0.99–1.02	0.68	1.00	0.97–1.02	0.70
Previous bladder cancer							
No	309	Ref	–	–	Ref	–	–
Yes	93	**1.91**	**1.29–2.82**	**0.001**	**1.75**	**1.12–2.74**	**0.01**
Hospital							
A	112	Ref	–	–	Ref	–	–
B	68	1.39	0.78–2.48	0.27	1.59	0.67–3.79	0.30
C	66	1.71	0.99–2.96	0.06	1.48	0.66–3.31	0.34
D	43	1.42	0.74–2.72	0.29	1.10	0.39–3.15	0.85
E	31	0.94	0.40–2.19	0.88	0.86	0.28–2.66	0.79
F	31	0.98	0.44–2.20	0.96	1.15	0.43–3.08	0.78
G	27	0.61	0.18–2.03	0.42	0.57	0.14–2.25	0.42
H	24	0.92	0.35–2.43	0.87	1.10	0.32–3.82	0.88

### Differences in surgical approach to the lower ureter by tumour location

The surgical approach to excision of the lower ureter varied significantly according to the location of the most distal tumour: the lower-end was managed endoscopically for 82.4% of renal pelvis/calyceal and upper ureteric tumours versus 27.8% of mid and lower ureteric tumours (*p* < 2.2e-16, Table S1). 43 patients (10.7%) were reported to have a positive surgical margin. Of those, 25 (58.1%) patients had their most distal tumour in the lower ureter, 4 (9.3%) in the mid ureter, 8 (18.6%) in the upper ureter and 6 (14.0%) in the renal pelvis/calyces. We assessed the association of lower-end surgical technique, T-stage, location of the most distal tumour and upper-end surgical technique with a positive surgical margin by logistic regression (and with treating hospital also included as a covariable in the model). On unadjusted analysis, lower ureteric tumour location, T3/T4 tumour stage, open upper-end surgical technique and transurethral dissection and pluck as management of the lower-end were significantly associated with positive surgical margin. Ureteric tumour locations and T3/T4 tumour were significantly associated with positive surgical margin on adjusted logistic regression (Table S2).

### Association of patient, tumour and surgical factors with secondary survival outcomes

Reviewing P-RFS, higher T-stages (T2, T3, T4 and Tis), upper and lower ureteric tumour location and patient age were associated with increased risk of extra-vesical pelvic recurrence on adjusted Cox-regression, although with 29 cases of such recurrence, confidence intervals were wide for many of these factors (Table S3). On adjusted analysis, higher T-stages (T2, T3, T4), node-positive disease and positive surgical margin were associated with M-RFS (Table S4), while higher T-stages (T2, T3, T4), node-positive disease, multifocal disease, not receiving mitomycin C post-operatively and higher age were associated with shorter OS (Table S5). On adjusted analysis, undergoing a laparoscopic extravesical excision was associated with worse CSS, along with higher T-stages (T2, T3, T4), node-positive disease and having a procedure at Centre G (Table S6).

## Discussion

Our results demonstrate that in appropriately selected patients, lower ureteric management technique does not affect our primary and secondary outcomes of intra-vesical or pelvic recurrence rates, metastases free survival or overall survival following NU. Interestingly a laparoscopic extravesical excision was associated with worse CSS, though the reasons for this are unclear. A prior ureteroscopy was however associated with a higher intra-vesical recurrence rate following NU, along with lower ureteric tumour location, previous bladder cancer and male gender. When all factors above were considered by adjusted Cox-regression, T-stage, N-stage and a positive surgical margin were associated with M-RFS. This suggests pathological factors drive the development of distant metastases, whereas tumour seeding (from manipulation in the form of ureteroscopy or from a more distal ureteric tumour) and unstable urothelium ( history of previous bladder cancer or multifocal ureteric disease) may lead to the development of intra-vesical recurrence.

In contrast to our results, several studies have demonstrated that the lower-end surgical technique does affect intra-vesical recurrence. A large meta-analysis confirmed that while there are some conflicting results between individual studies, an extra-vesical approach resulted in a higher rate of intra-vesical recurrence than either endoscopic or trans-vesical excision [23]. Importantly, tumour location was less well characterised in that study and this and other confounding factors may have biased the analysis. In contrast to the meta-analysis results, Walton et al. and more recently the ROBUUST collaboration reported that endoscopic approaches or transurethral resection of the bladder cuff respectively were associated with increased risk of intra-vesical recurrence [7, 12]. However, across individual studies there was little heterogeneity in lower-end techniques with 82.5% undergoing a ‘pluck’ in the ROBUUST study, and only 11.6% undergoing endoscopic approaches in the study by Walton et al. Encouragingly, the recently published study by Veeratterapillay et al. [4] found lower-end surgical technique was not associated with intra-vesical recurrence-free survival, although lower-end technique description was limited to endoscopic assisted (67.5% cases), open excision with bladder cuff (28.7% cases) and robotic excision (3.8% cases) with no further details. We present a large multicentre series including a spread of lower-end techniques which are well described to allow full interpretation in the context of disease location and stage. As well as the lower-end technique, we reassuringly demonstrated the consistent finding that tumour location, history of bladder cancer and male gender were predictors of intra-vesical recurrence [23].

The impact of ureteroscopy on intra-vesical recurrence rate following NU has been debated. We add to the growing evidence that pre-NU diagnostic ureteroscopy increases the likelihood of intra-vesical recurrence. Unfortunately, we do not have data on whether a ureteroscopic biopsy was performed or whether a stent was left in situ, which may have a greater impact than ureteroscopy alone [24]. Furthermore, the relative value of ureteroscopy was not assessed in this or previous studies and the risk–benefit needs to be clearly considered.

A positive surgical margin could be considered the result of advanced disease or because of surgical technique. While a positive surgical margin was not associated with an increased risk of intra-vesical recurrence in our cohort, it did adversely affect metastatic-free survival. Our study reports a positive surgical margin rate of 10.7%. To understand the interplay between positive surgical margin and lower-end surgical technique, in the context of tumour stage and upper-end surgical technique, we undertook post-hoc logistic regression analysis. By this method, we demonstrated that a ureteric tumour location and T3/T4 tumour stage were associated with positive surgical margin, although reassuringly upper end and lower ureteric management techniques did not affect this outcome.

As per Katims et al. [16], perioperative intravesical chemotherapy was not found to be protective against bladder recurrence in this cohort. This is in disagreement with previous RCTs [17, 25]. Despite RCTs being published in 2011 and 2013, only 19.7% in the current series received adjuvant intravesical chemotherapy (26.3% in the Katims et al. study [16]). Our study (and that of Katims et al.) were therefore potentially underpowered to assess the benefit from neoadjuvant chemotherapy on intra-vesical recurrence. The reasons for the lack of utilisation of mitomycin are unclear, though this varied by unit (with one using in 84% patients, and two using in none). Of particular note, while Mitomycin C was not associated with a reduction of disease recurrence (local or distant) in our cohort, it was associated with better overall survival. We speculate that either healthier patients were being selected to receive treatment (either actively by the surgical teams, or passively as they were more likely to recover well from their NU and be deemed fit for mitomycin C), or receiving Mitomycin C was a marker of a patient receiving care which best followed current guidelines.

Given the retrospective nature of this study there are a number of limitations. It was not possible to assess the decision-making on the surgical approach for each individual patient. Surgical technique did appear to vary significantly based on the location of the most distal tumour, suggesting surgeons treating tumours of the distal and mid ureter are less likely to opt for an endoscopic approach in these cases because of a perceived risk of poorer outcomes. In addition, data were not collected regarding patient co-morbidities or general fitness and thus the contribution of factors such as hypertension, diabetes and chronic kidney disease to increased intra-vesical recurrence could not be confirmed. Furthermore, we had insufficient data on smoking history to include this as a variable in the Cox regression analysis. Extra-vesical pelvic recurrence was relatively rare in this cohort (29 cases) and hence the study was underpowered to assess the association of factors with pelvic recurrence as a single entity (confidence intervals were wide in the Cox regression models). Despite these limitations, the study represents a real-world assessment of practise across Scotland, and includes a more heterogeneous sample of management techniques for the lower ureter than in previous studies. While an RCT is required to demonstrate definitively the superiority (or inferiority) of particular techniques, the current study suggests surgeons are appropriately selecting management technique with their patients.

## Conclusions

These data demonstrate that in appropriately selected patients, lower ureteric management technique does not affect tumour recurrence rate. Diagnostic ureteroscopy was associated with a higher recurrence rate following NU and the indication for this should be carefully considered. Positive surgical margin was associated with worse secondary outcomes, and the data presented above suggest that ureteric tumours and those with T3 or T4 disease were at greatest risk. The surgical approach in these cases should be carefully considered.

## Supplementary Information

Below is the link to the electronic supplementary material.

Supplementary file1 (XLSX 38 KB)

Supplementary file2 Differences in B-RFS according to lower-end surgical technique assessed by KM (JPG 364 KB)

## Data Availability

Data are available for bona fide researchers who request it from the authors.

## References

[1] Rouprêt M, Babjuk M, Burger M, Compérat E, Gontero P, Liedberg F, Masson-Lecomte A, Mostafid AH, Palou J, van Rhijn BWG, Shariat SF, Sylvester R. (2022) Upper Urinary Tract Urothelial Cell Carcinoma. EAU Guidelines. Edn. presented at the EAU Annual Congress Amsterdam

[2] Siegel RL (2021). Cancer Statistics, 2021. CA Cancer J Clin.

[3] Favaretto RL (2010). Comparison between laparoscopic and open radical nephroureterectomy in a contemporary group of patients: are recurrence and disease-specific survival associated with surgical technique?. Eur Urol.

[4] Veeratterapillay R et al. (2021) Ten-year survival outcomes after radical nephroureterectomy with a risk-stratified approach using prior diagnostic ureteroscopy: a single-institution observational retrospective cohort study. BJU Int 10.1111/bju.15627 34726325

[5] Margulis V (2009). Outcomes of radical nephroureterectomy: a series from the Upper Tract Urothelial Carcinoma Collaboration. Cancer.

[6] Brown HE, Roumani GK (1974). Conservative surgical management of transitional cell carcinoma of the upper urinary tract. J Urol.

[7] Walton TJ (2011). Oncological outcomes after laparoscopic and open radical nephroureterectomy: results from an international cohort. BJU Int.

[8] Ni S (2012). Laparoscopic versus open nephroureterectomy for the treatment of upper urinary tract urothelial carcinoma: a systematic review and cumulative analysis of comparative studies. Eur Urol.

[9] Blackmur JP (2015). Matched-pair analysis of open versus laparoscopic nephroureterectomy for upper urinary tract urothelial cell carcinoma. Urol Int.

[10] Veccia A (2020). Robotic versus other nephroureterectomy techniques: a systematic review and meta-analysis of over 87,000 cases. World J Urol.

[11] Huang YP et al. (2022) Is robotic superior to laparoscopic approach for radical nephroureterectomy with bladder cuff excision in treating upper urinary tract urothelial carcinoma?. J Endourol 10.1089/end.2022.0154 36267017

[12] Veccia A (2022). Robotic vs Laparoscopic nephroureterectomy for upper tract urothelial carcinoma: a multicenter propensity-score matched pair "Tetrafecta" analysis (ROBUUST Collaborative Group). J Endourol.

[13] Li WM (2010). Oncologic outcomes following three different approaches to the distal ureter and bladder cuff in nephroureterectomy for primary upper urinary tract urothelial carcinoma. Eur Urol.

[14] Xylinas E (2014). Impact of distal ureter management on oncologic outcomes following radical nephroureterectomy for upper tract urothelial carcinoma. Eur Urol.

[15] Attalla K (2019). Management of distal ureter and bladder cuff at the time of nephroureterectomy: surgical techniques and predictors of outcome. Future Oncol.

[16] Katims AB (2021). Risk factors for intravesical recurrence after minimally invasive nephroureterectomy for Upper Tract Urothelial Cancer (ROBUUST Collaboration). J Urol.

[17] O'Brien T (2011). Prevention of bladder tumours after nephroureterectomy for primary upper urinary tract urothelial carcinoma: a prospective, multicentre, randomised clinical trial of a single postoperative intravesical dose of mitomycin C (the ODMIT-C Trial). Eur Urol.

[18] Team RC (2018) R: A /language and environment for statistical computing. R Foundation for Statistical Computing, Vienna, Austria.; Available from: http://www.R-project.org/

[19] Team R (2016) RStudio: integrated development for R. RStudio, Inc., Boston, MA. Available from: http://www.rstudio.com/

[20] Therneau TM (2015) A package for survival analysis in S, Version 2.38. Available from: https://CRAN.R-project.org/package=survival

[21] Kassambara AK, Survminer M (2019) Drawing survival curves using ‘ggplot2’ R package version 0.4.4. Available from: https://CRAN.R-project.org/package=survminer

[22] Harrison ED, Ots TR (2020) Finalfit: quickly create elegant regression results tables and plots when modelling. [R package]; version 1.0.2:[Available from: https://CRAN.R-project.org/package=finalfit

[23] Seisen T (2015). A systematic review and meta-analysis of clinicopathologic factors linked to intravesical recurrence after radical nephroureterectomy to treat upper tract urothelial carcinoma. Eur Urol.

[24] Chung Y (2020). Impact of diagnostic ureteroscopy before radical nephroureterectomy on intravesical recurrence in patients with upper tract urothelial cancer. Investig Clin Urol.

[25] Ito A (2013). Prospective randomized phase II trial of a single early intravesical instillation of pirarubicin (THP) in the prevention of bladder recurrence after nephroureterectomy for upper urinary tract urothelial carcinoma: the THP Monotherapy Study Group Trial. J Clin Oncol.

