# Supplementary figures and images for: Assessment of association between lower ureteric excision technique and oncological outcomes for upper urinary tract urothelial carcinoma: retrospective analysis from the Scottish Renal Cancer Consortium
Source: World J Urol. 2023 Jan 24;41(3):757–65. doi: 10.1007/s00345-023-04283-5 (PMC10082697; doi:10.1007/s00345-023-04283-5)

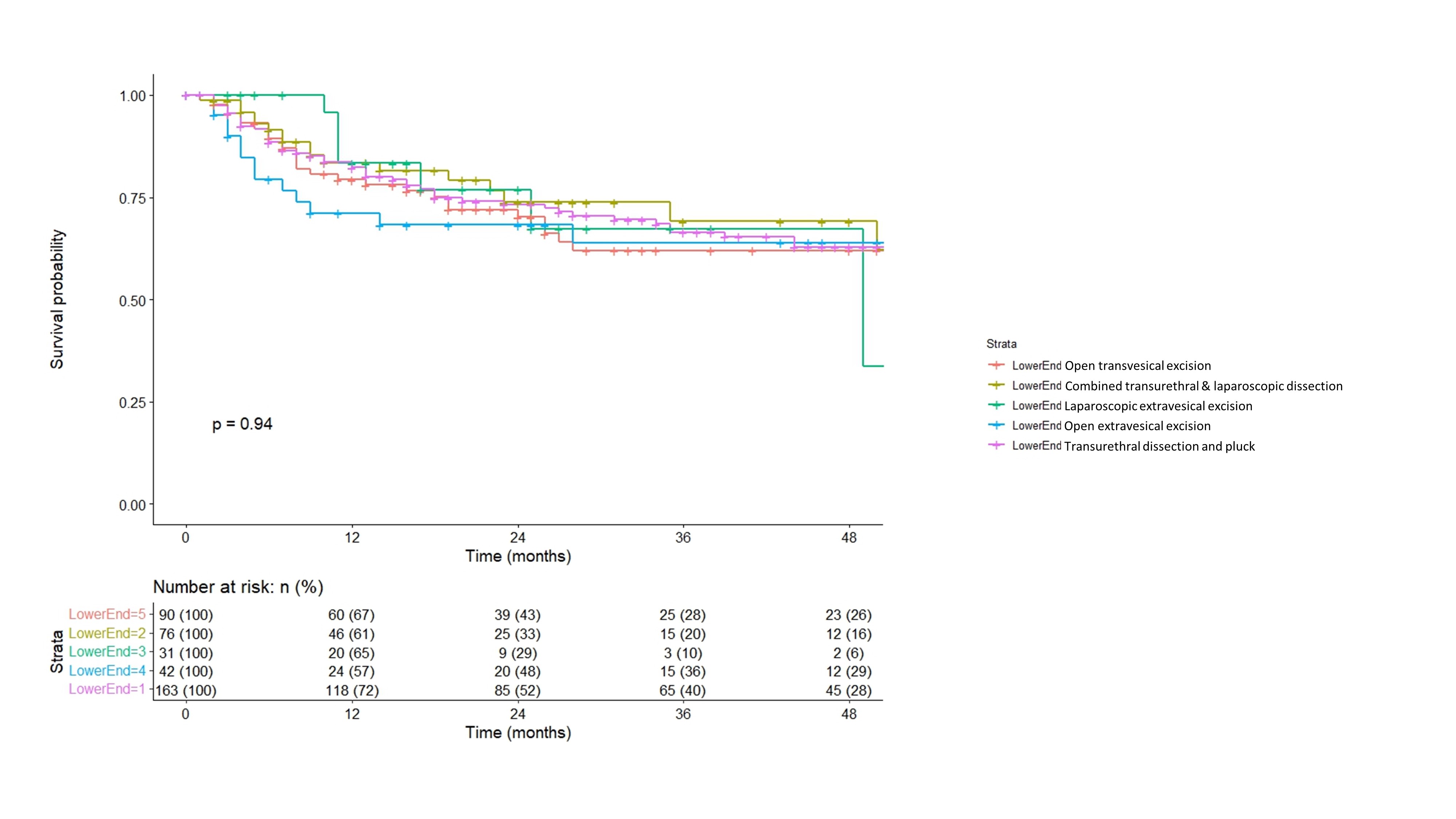

Supplement: Supplementary file 2 — Supplementary file2 Differences in B-RFS according to lower-end surgical technique assessed by KM (JPG 364 KB) [file 345_2023_4283_MOESM2_ESM.jpg]
